# Abstracts

**Published:** 1980

**Authors:** 


### Abstracts

### South West Surgical Club
Autumn Meeting 1980, Bristol

The South West Surgical Club was particularly pleased to welcome Professor Franchini as a Guest of Honour in a return visit, following a very successful visit of the Club to his clinic in Bologna in 1979. An abstract of his paper appears below.

### MULTIPLE TUMOURS OF THE LARGE BOWEL

**A. Francini and R. Giardino, Bologna**

It now seems to be definitely proved that in any patient who has had a carcinoma the risk of developing a second tumour is greater than would be expected by chance alone. This is particularly true in the bowel.

True multiplicity occurs when different tumours are completely distinct and this can be due to local susceptibility manifesting itself in different areas at the same time, or to malignant transformation of pre-existing benign lesions such as polyps or ulcerative colitis.

It has been calculated that the predisposition to a second tumour is ten times greater than the first and the probability of a third tumour is even greater. 3% are multiple at the time of discovery and another 3–4% of those who survive the first tumour must expect a second. This probability is doubled when a tumour is accompanied by polyps and in these cases it is the authors' practice to perform total colectomy.

The greatest risk of recurrence is in the first two years but cases have been recorded up to fifteen years. Follow up is very important and patients who have survived surgery for colonic cancer should be kept under continuous control for the rest of their lives and should be seen at least annually. When such a patient develops a second tumour the second operation should be a total colectomy.

### RETURN TO WORK AFTER OPERATIONS

**J. Meyrick Thomas, Exeter**

In an attempt to investigate when patients return to work after operations, 90 patients between the ages of 20 and 60 having operations under general anaesthesia over a 2-month period were given questionnaires to take home with them and to complete question by question as their convalescence progressed. The questionnaire (which had an 80% response rate) asked for the dates of return to feeling normal, normal appetite, sexual activity, exercise and work. These average times taken to resume normal wellbeing, exercise and sexual activity correlated very well (correlation coefficient 0.88) with return to work.

This correlation represented all operations performed under general anaesthesia: although most rapid recoveries (less than a week) were of patients having minor 'day-case' operations there was considerable overlap between major and minor operations, and it was apparent that no particular operation had a predictable length of convalescence. It is suggested that patients should be advised to return to work when they have recovered in a more general sense (as indicated by return to normal exercise, wellbeing and sexual activity) rather than after a specific interval. Such a policy might encourage the less motivated to return to work earlier than they do at present.

### IMAGE INTENSIFICATION CHOLANGIOGRAPHY

**P. A. Farrands, Bath**

Despite improvements in the technique of per-operative cholangiography, the procedure remains *costly, time consuming* and in some instances *inaccurate.*

In an effort to overcome these problems we have for the past three years employed a mobile image intensifier to screen the common bile duct during cholangiography. As well as cutting the cost and eliminating the time taken for film development, this technique allows the common bile duct to be screened through-out its length before focusing on any suspicious areas with a magnifying facility.

One hundred and twenty six patients undergoing cholecystectomy for cholelithiasis have been studied prospectively over an eight month period.

In one hundred and seven patients the image intensifier cholangiogram was reported as normal. Nineteen patients had an abnormality. In sixteen of these cases gall stones were found in the common bile duct; in one case biliary sludge was present, and in two patients, narrowing of the common bile duct was present with proximal dilatation. No 'false positives' occurred, and every patient with an abnormality on cholangiography had an abnormality on exploration of the common bile duct. Six months after surgery, all patients were asymptomatic.

Skillings (1979) calculated an extra thirty minutes was added to the operation of cholecystectomy, and that each film cost approximately 60 pence excluding the developers and staff's salary. The average time taken for per operative image intensification after cannulation of the cystic duct was three minutes in our hands, and no cassettes were used unless films were required for a permanent record. 'False positive' cholangiograms often result from inexperienced interpretation of the adynamic X-ray pictures. Per operative cholangiography with an image intensifier allows a direct dynamic study of the biliary tree with infusion of variable amounts of Hypaque. Magnification is available and films may be obtained when required for a permanent record.

As judged by our results, we feel that image intensification cholangiography is accurate, safe, speedy and cheap and recommend it unhesitatingly as a standard method of delineating the biliary tree.

### MANAGEMENT OF NEONATAL DIAPHRAGMATIC HERNIAE

**N. J. Barwell, Truro**

Failure of closure of the pleuro-peritoneal canal with herniation of most of the abdominal contents into the chest has been recognised for many years. The Perinatal Mortality Survey showed that it accounted for many stillbirths and deaths within the first hour of life. Many more cases are still dying within the first twenty-four hours and it is my belief that many of these can be salvaged. A survey in the South West between 1943 and 1974 showed a high proportion of cases with no other abnormality. In the last five years, all cases diagnosed in Truro have been subjected to early surgery through an abdominal incision. Six out of nine cases reaching the operating theatre have survived. The cases dying all had serious lethal other congenital abnormalities. Because these infants travel badly, I believe that any Surgeon with access to the facilities of a Special Care Baby Unit could operate on these technically straight forward cases and in this way, normal healthy individuals can be salvaged.

### AORTIC VALVE REPLACEMENT
### Current Methods and Results

**N. V. Mandke, D. Prakash, J. D. Wisheart, Bristol**

**SUMMARY**

Between April 1978 and July 1980, 54 patients had aortic valve replacement using cold potassium cardioplegia to protect the myocardium. The mean age was 52.8 years. 40 patients had calcific aortic stenosis and the remaining had aortic incompetence. The cardiopulmonary bypass time was 1 hour 50 minutes. The mechanical activity of the heart was arrested by injecting the cold cardioplegic solution into the coronary arteries and the mycardial temperature was maintained at 10°C – 20°C by topical cooling. The technique of cold cardioplegia protects the myocardium from ischaemic damage. 35 biological tissue valves and 19 prosthetic valves were inserted using interrupted sutures. There were 3 late deaths. 44 patients had an uneventful recovery. The average stay in intensive care was 30 hours.

During the short follow up periods 46 of the 51 survivors were symptom free and 43 had returned to full time work. 15 patients were on cardiac drugs and 18 who had prosthetic valves were on anticoagulants.

In conclusion, these results confirm that:

1. Cardioplegia protects the myocardium and provides good operating conditions.
2. Operative and late mortality is low.
3. These patients resume active life, after recovery from the operation.

### A NEW OPERATION FOR REPAIR OF COMPLETE RECTAL PROLAPSE IN THE ELDERLY

**H. J. Espiner, Bristol**

A new operation has been devised for the cure of complete rectal prolapse in the elderly, often infirm patient who would not easily withstand abdominal surgery.

The operation is carried out under general anaesthesia with the patient prone and the buttocks strapped apart. From the bed of the excised coccyx the rectum is mobilised and the grossly redundant bowel thus delivered and plicated. Through the same exposure the margins of the levator ani are approximated behind the rectum so advancing the anorectal junction towards the pubis and accentuating the angle the rectum makes with the anal canal. The plicated rectum is sutured to the reconstructed levator ani and the wound closed with suction drainage.

Over the last ten years some 23 patients have undergone this procedure. 22 of these were females and over 75% of them were 75 years or older. 15 patients are available for assessment of the result at an average of 1.5 years post-operatively. 14 have been completely cured of the prolapse and continence has been restored in three. There has been an improvement of continence in a further four cases.

Because this is a relatively minor procedure, minimal disturbance to the patient and her alimentary function it could find a useful place in the management of complete rectal prolapse in elderly women.

### A COMPARISON OF TWO APPROACHES TO THE PROBLEM OF MANAGING ACUTE REJECTION IN RENAL TRANSPLANT RECIPIENTS

**B. Pentlow, Bristol**

Whilst the search for new immuno-suppresive agents continues, our knowledge of the optimal methods of using existing drugs is sparse.

This trial compares the results of two approaches to the problem of managing acute rejection.

A 'Conventional' group of 31 patients received standard maintenance doses of Prednisolone and Azathioprine; when acute rejection was diagnosed it was treated with 3 grams of intra-venous Methyl-Prednisolone.

The 'Experimental' group of 31 patients received similar drugs, but in addition received high 'anti-rejection' doses of oral Prednisolone whether or not acute rejection occurred. The dosage was 200 mg. Prednisolone on day 3 post-operatively, tapering down to 40 mg. after 2 weeks. down to 40 mg. after 2 weeks.

Results were assessed at 3 months for patient survival, graft survival or morbidity. (Three months comparisons are valid as the majority of grafts lost due to rejection are lost in the first few weeks after transplantation.)

**RESULTS**

**Death**

Conventional Group – 1
Experimental Group – 0

**Rejected Grafts**

Conventional Group – 12 grafts lost
Experimental Group – 3 grafts lost

**Morbidity**

The incidence and severity of steroidal facies was somewhat higher in the experimental group.

The incidence of wound infection and secondary haemorrhage was greater in the conventional group reflecting complications after the removal of rejected kidneys.

The incidence of other morbidity was similar.

**CONCLUSIONS**

The results of both groups are acceptable. We have guarded optimism that the results of the experimental group at three months will be maintained.

### RESULT OF A RANDOMISED TRIAL TO COMPARE LOCAL WITH GENERAL ANAESTHESIA FOR INGUINAL HERNIA REPAIR

**C. Teasdale, R. E. Horton, A. McCrum and B. Williams, Bristol**

The interim results were presented on 77 unselected patients already entered into a prospective randomised controlled trial to compare local regional anaesthesia (LA) with general anaesthesia (GA) for repair of unilateral, clinically reducible inguinal herniae. Forty patients had LA and 37 GA, both groups being equally matched for age, obesity and types of hernia. Post-operative subjective symptoms were measured using linear analogue self assessment questionnaires.

The operation was concluded successfully in all patients having LA – the use of intravenous sedation during the procedure and the presence of an anaesthetist being found unnecessary. LA was acceptable to 85% of those patients in whom it was used, such that they would agree to its use again – even though the majority experienced mild pain during the operation. There were, however, significant post-operative symptoms of sore throat, headache, nausea and vomiting in the GA group, compared to those having LA, but the degree of pain, limitation of mobility and analgesic requirements were similar in both groups.

Two patients in each group were found to have irreducible herniae at operation. More than 90% of patients were discharged 24 hours post-operatively, however, in both groups approximately 30% thought that this was too early. Five patients having LA and two patients having GA called their general practitioner during the first week after discharge.

No category of consenting patient or hernia was found to be unsuitable for LA, although obesity and sliding or irreducible herniae were factors which made the operation more difficult and time consuming than it would have been with GA.

### LONG DACRON GRAFTS IN THE TREATMENT OF CRITICAL ISCHAEMIA

**R. E. Horton, Bristol**

The long graft is defined in this paper as a 10 mm dacron graft from the common iliac artery or the aorta to the distal popliteal. In this presentation the indication for the operation was combined iliac and femoral artery disease. Twenty four grafts have been done in 21 patients with an initial mortality of 5%.

The operation is described.

Studies with a pulse volume recorder show that a much more dramatic result can be achieved with this operation than with the ilioprofunda graft which is the preferred operation of most vascular surgeons in this country. The clinical result is also much better.

Follow up studies with a pulse Doppler imaging system (MAVIS GEC Medical Ltd) show no calibre change in the first 4 months. In later studies there was a progressive tapering of the distal third which is thought to be the haemodynamic result of joinig a 10 mm graft to a much narrower popliteal artery.

Evidence to support the use of a graft of 10 mm is produced.

Kinking may occur in dacron grafts which pass the knee joint. Studies are described and prevention discussed.

Using the life table method of analysis patency was 79% at four years.

### BLEEDING OSEOPHAGEAL VARICES – SHUNT OR STAPLE?

**R. N. Baird and M. H. Thompson, Bristol**

28 consecutive operations for bleeding oesophageal varices were performed in 3½ years in the Surgical Unit, Bristol Royal Infirmary from 1977–80. The diagnosis in one third (9) was alcoholic cirrhosis, and the remainder included chronic active hepatitis, post-hepatitic cirrhosis and primary biliary cirrhosis. Non-cirrhotic causes included extra-hepatic portal vein occlusion and 'minimal change' liver histology. Two thirds of the patients were over 50 years of age. Those operated on were selected from a much larger group who were treated medically. The continuous intravenous infusion of 0.5 units/min of pitressin was more effective in controlling bleeding than bolus infusion. The indication for operation in 20 patients was uncontrolled bleeding (15-30 units of blood transfused in 48 hours) and repeated smaller bleeds in the remainder. The direction of blood flow in the portal vein was used to select the type of operation. There were 11 dacron portacaval H-grafts and 9 conventional portacaval shunts. The remainder underwent a proximal or distal splenorenal (Warren) shunt or oesophageal stapling.

81% (21 of 26) patients left hospital alive. The distal splenorenal shunt did not control bleeding on 2 occasions and revision operations (oesophageal stapling; portacaval shunt) were successfully done. Five hospital deaths were caused by liver failure (4) and bleeding (1).

These results show that a portacaval shunt or H-graft will effectively control massive haemorrhage if patients have adequate hepatic reserve.

### PANCREATIC TRAVELS IN THE AMERICAS

**R. C. N. Williamson, Bristol**

### CONTROVERSIES IN CARE: A TRANSATLANTIC PERSPECTIVE

**A. E. B. Giddings, Bristol**

### THE MANAGEMENT OF PEYRONIE'S DISEASE

**David Kirk and Patrick Smith, Bristol**

The Medical and surgical management of Peyronie's disease were discussed. No medical treatment has been proved of undoubted value in this condition, a view to which the disappointing experience with ultrasonic therapy in Bristol confirms. Once the acute phase has resolved, many patients will be able to resume intercourse without further treatment. For the few in whom residual deformity remains a problem, a variation of Nesbit's operation of contralateral correction has recently been suggested (Pryor and Fitzpatrick 1979). The technique was described and experience of the use of the apparatus in 6 patients reviewed. In 5, good correction of the deformity was obtained; the sixth was able to resume satisfactory intercourse. Two patients were unable to achieve penetration because of flaccidity distal to the plaque. One of these had a Finney intracorporial impotence prosthesis inserted with satisfactory results. Nesbit's operation appears to be a satisfactory and straightforward technique for patients with persisting and troublesome chordee due to Peyronie's disease.

### PRIORITIES IN FACIAL INJURIES

**J. W. Ross, Bristol**

Although one in every eight hundred motorcyclists in this country is killed every year, there are a high number of non-fatal maxillo-facial injuries resulting from all kinds of road traffic accidents. These injuries would be reduced by an estimated figure of 100,000 in ten years if seat belts became compulsory.

Facial injuries are commonly associated with head injury and the latter, of course, takes priority in treatment. Also high on the list of priorities are eye injuries, intra-abdominal bleeds, chest injuries and open joints. The facial fractures are dealt with under the same anaesthetic wherever possible and if time allows, this treatment is always definitive. Bleeding from facial fractures is very rarely an indication for urgent treatment and shock is unusual in cases with facial injuries alone.

Most delays in treatment are due to head injuries.

If there is any doubt about the patency of the airway pre or post operatively, a tracheostomy is performed, particularly in cases of head injury.

Leak of cerebrospinal fluid is not an urgent problem, but precautions should be taken to prevent meningitis, such as prophylactic antibiotic, resting of the patient and avoidance of nose blowing.

### ENDOSCOPIC AND RADIOLOGICAL ASSESSMENT OF VELOPHARYNGEAL INCOMPETENCE

**R. W. Pigott, Bristol**

Inadequate correction of congenital incompetence of the velopharyngeal isthmus has serious consequences for the development of normal speech intelligibility, ranging from total incomprehensibility to an abnormal tone of voice for which the patient is teased and becomes reluctant to speak and fails to develop. Treatment has tended to be empirical, one of many available operations being practiced routinely without consideration of the specific problems involved.

Over the last ten years a system has been developed at Frenchay Hospital, Bristol for the combined recording of an endoscopic image of the velopharyngeal isthmus, viewed from the nasal side during connected speech, with a lateral pharyngeal view using an image intensifier. The two images are combined using a split screen unit developed by Mr. A. P. W. Makepeace in the University of Bristol, recorded on video tape and displayed on a monitor.

The system provides information on the shape and size of the isthmus at rest and during speech and a crude measurement of the size of the defect is obtained. Those muscle groups primarily responsible for movement are noted.

With this information a logical selection of operation has become possible and criteria for success have been more clearly defined. The success of operations based on these investigations have risen from figures usually quoted in the literature around 60% to over 80%.

### MICROVASCULAR SURGERY IN THE PLASTIC SURGERY UNIT AT FRENCHAY

**Paul Townsend, Bristol**

With the advent of the microscope, it is now possible to operate on vessels from about 1 mm upwards. The first application in plastic surgery was to re-implant digits. Subsequently, with greater knowledge of anatomy, it became possible to isolate feeding vessels in areas of skin, e.g. in the groin flap, enabling it to be transferred and reconnected to suitable recipient vessels. This concept has now been taken further to isolate segments of bone nerves and tendons with their blood supply and re-establish them in new sites. A number of examples were demonstrated with results often superior to and achieved in much less time than traditional methods. At the present time some thirty-six cases have been carried out at Frenchay, of which about half were cases resulting from lower limb injury.

In compound fractures of the lower third of the tibia, cross leg and muscle flaps are either impossible or very difficult. With a free flap, it is possible in one operation to re-surface the bone, with about one months stay in hospital. In the past, tube pedicles were often raised requiring up to six months in hospital and at least five operations. A microvascular procedure takes eight to twelve hours and this means that total operating time is often reduced as well as hospital stay.

